# Audiovisual estimation of Time-to-contact

**DOI:** 10.3758/s13414-025-03176-6

**Published:** 2026-01-13

**Authors:** Solène Leblond, Robin Baurès, Julien Tardieu, Céline Cappe

**Affiliations:** 1 CerCo (Centre for Brain and Cognition Research), CNRS UMR 5549, University of Toulouse, 118 Route de Narbonne, 31062 Toulouse, France; 2 Maison des Sciences de l’Homme de Toulouse, CNRS UMR3414, University of Toulouse, 5 Allée Antonio Machado, 31100 Toulouse, France

**Keywords:** Attentional capture, Attention: Selective, Visual search

## Abstract

Time-to-contact (TTC) is the remaining time for a moving object to reach its observer. A good estimation of TTC is essential in everyday situations such as crossing a road or catching a ball. So far, most studies have only looked at estimation of TTC at constant speed, and in visual condition, whereas it is a multisensory task by essence. In our study, we investigated TTC estimation at constant or accelerated speed for three modalities: Auditory, Visual and Audiovisual. At constant speed, it has been demonstrated that TTC estimation performance is already accurate so the addition of auditory cues would not lead to significant performance changes. However, at accelerated speed, visual estimation of TTC becomes more impaired. In this context, auditory cues were expected to play a more prominent role in improving performance. For this reason, we hypothesized that V and AV performance would be identical at constant speed, whereas at accelerated speed, auditory cues would allow a better performance in AV compared to V. Our results show that observers do use both modalities in the AV condition and therefore demonstrate a multisensory integration, but for both levels of acceleration and better performance is not always observed in the multimodal condition. Specifically, auditory cues lead to an underestimation of TTC, which compensates for the visual overestimation. Therefore, whether multisensory integration has a beneficial or detrimental effect on the performances of TTC estimation depends on the level of baseline error in the visual-only and auditory-only conditions.

**Supplementary Information:**

The online version contains supplementary material available at 10.3758/s13414-025-03176-6.

## Introduction

In our daily lives, we are surrounded by moving objects that we want to avoid or, on the contrary, to collide with. For this, the ability to determine the remaining time before the object reaches its observer is crucial. This time corresponds to the time-to-contact (TTC) of the moving object. For example, during a sport activity, to hit or to catch a ball, it is necessary to correctly estimate the TTC of the ball. Daily, when crossing a road, a pedestrian must estimate the TTC of approaching vehicles to cross safely. The same question arises when a driver has to brake to avoid colliding with a vehicle in front of him. In general, every journey involves estimating if and when objects in our environment might collide with us; this time-to-contact estimation is therefore crucial for autonomous travel.

Numerous studies have focused on the performance of TTC estimation, with some of these studies using a prediction motion task (PM). In this paradigm, a moving object is occluded during its trajectory and the observer has to estimate its arrival time (Baurès et al., 2018; Bennett & Benguigui, 2016; Feldstein, 2019). The results of the PM task are well documented. In particular, some of these studies demonstrated a linear relationship between the TTC of the object and the time estimated by the participant (Benguigui et al., 2008). In the studies demonstrating a linear relationship, it is often, although not always, found a slope lower than 1 combined with a positive intercept. These values lead the participants to overestimate the TTC (the participant perceives the ball as arriving later than it does) for short TTC whereas they underestimate the TTC (the participant perceives the ball as arriving earlier than it does) for long TTC. It however remains to be determined if the transition point from over- to underestimation is around 1 s (e.g., Bennett et al., 2010) or influenced by the mean of the different TTC levels used in the task (e.g., Battaglini & Mioni, 2019).

We encounter stimuli from different sensory modalities in our environment. It has been widely demonstrated that the brain integrates this information together, creating multisensory integration (Stein & Meredith, 1993). This integration is the basis for performance improvements in everyday tasks, particularly in the processing of motion (Soto-Faraco et al., 2023). It reduces reaction time and/or increases the rate of correct responses. In their study, Cappe et al. (2009) reveal selective integration of multisensory looming stimuli. Participants had to detect looming or receding motion in visual (V), auditory (A) or audiovisual (AV) modalities as quickly as possible. Performance was significantly improved for looming stimuli. Under natural conditions, two sensory modalities could be used to estimate TTC: the V and the A modalities. However, the literature on auditory perception of TTC is very sparse (Gray, 2011). Even fewer studies have examined multisensory AV integration in this task, and their authors remain divided. Most of these studies do not show a clear advantage for the contribution of auditory information when the object is presented in AV (DeLucia et al., 2016; Hassan & Massof, 2012; Keshavarz et al., 2017; Prime & Harris, 2010; Zhou et al., 2007). In fact, there appears to be no difference between the V and AV conditions, which has been interpreted as a "winner-take-all" mechanism (Lee et al., 1999). Some of these studies also measured the relative weights of A and V cues, using a behavioral reverse-correlation approach (DeLucia et al., 2016; Keshavarz et al., 2017). Although V and AV TTC estimation performances were identical, they showed a use of auditory cues during AV TTC estimation, although V cues remained predominant. Oberfeld et al. (2022) compared the TTC estimation in A and AV modalities of two vehicle types, varying the loudness level using novel interactive audiovisual virtual-reality system for traffic scenarios. For an identical TTC, varying loudness level leads to changes in estimation performance in both A-only and AV, showing that audiovisual TTC estimates are to a significant extent based on the loudness of the approaching object. These findings are in favour of using an auditory cue when estimating TTC. It is crucial to note that most of these studies testing the AV context mainly used stimuli moving at a constant speed, for which the visual perception of TTC is relatively good. According to the principle of inverse effectiveness (Stein et al., 2009), the benefit of a multisensory condition is inversely proportional to the performance of monosensory conditions. In other words, there would be room for the auditory information to contribute to the TTC estimation only if the visual information were poorly used. Literature suggests that when the object is accelerating, observers do not take this acceleration into account (Benguigui & Bennett, 2010; Watamaniuk, 1992; Werkhoven et al., 1992), and estimate the TTC based on motion at constant speed. The result of this failure to account for acceleration is that TTC is overestimated for accelerating objects (the participant responds too late) and underestimated for decelerating trajectories (the participant responds too early). It has been demonstrated that acceleration is well perceived in the auditory domain (Carlile & Leung, 2016; Lutfi & Wang, 1999). It therefore appears that observers should benefit from the AV context when the object accelerates. Our hypothesis is as follows: at constant speed, observers correctly estimate the TTC of the approaching object based on the visual cues, so the addition of auditory cues and multisensory integration would not lead to significant performance changes. However, at accelerated speed, visual estimation of TTC becomes more impaired. In this context, auditory cues are expected to play a more prominent role in estimating the TTC and therefore improving performance, in the AV condition compared to the V condition.

The study of Wessels et al. in (2022b) confirmed this theory. Using a virtual reality environment, they used a PM task to compare the performance of TTC estimation in V and AV for vehicles travelling at constant and accelerated speeds. In V-only, they observe a large overestimation of the TTC, increasing as the actual TTC increases. In AV, the addition of auditory cues corrected this overestimation pattern and provided a more accurate estimate, confirming the importance of auditory cues in detecting acceleration. That study however, did not test an A-only condition which would allow to better situate the AV performance in particular in the accelerated case. In a second study, Wessels et al. (2022a) compared audiovisual TTC estimation of three types of vehicles: internal combustion engine vehicles (ICEV), electric vehicles (EV) and EV with acoustic vehicles electric system (AVAS). The sound of ICEVs provided auditory cues specifically linked to acceleration as the engine noise varied significantly with changes in speed. In contrast, EVs are considered to be silent, their acceleration does not generate pronounced auditory variations. The AVAS system added an acoustic signature during acceleration, but the variation in sound was less pronounced than that of ICEVs, potentially making acceleration cues less perceptible. At constant speed, performance was identical for the three types of vehicles. At accelerated speed, the overestimation of TTC for EVs (with and without AVAS) suggested that participants did not fully integrate acceleration information into their judgments. For ICEVs, on the other hand, the TTC estimation performance was more accurate, showing that acceleration has been considered. The higher the sound level, the more the variation in sound induced by acceleration facilitated better TTC estimations, suggesting that participants used sound to perceive acceleration and improve their performance. However, in this study they did not test the performance obtained for visual-only estimation.

The aim of our study is to compare the TTC estimation performances in the three modalities A, V and AV, when the object moves at constant or accelerated speed, in order to observe a possible benefit of audio-visual multisensory integration.

## Materials and methods

### Participants

Twenty students (mean age = 24.2 years; SD = 3.1 years; range = 19–30 years; 13 women) were recruited. All participants had normal or corrected-to-normal vision, and were healthy and without any known oculomotor abnormalities. They participated after giving informed consent. Participants were naïve with respect to the purpose of the experiment. The experiment received the appropriate ethical authorization from a local ethical committee.

### Apparatus and experimental procedure

**Fig. 1:**
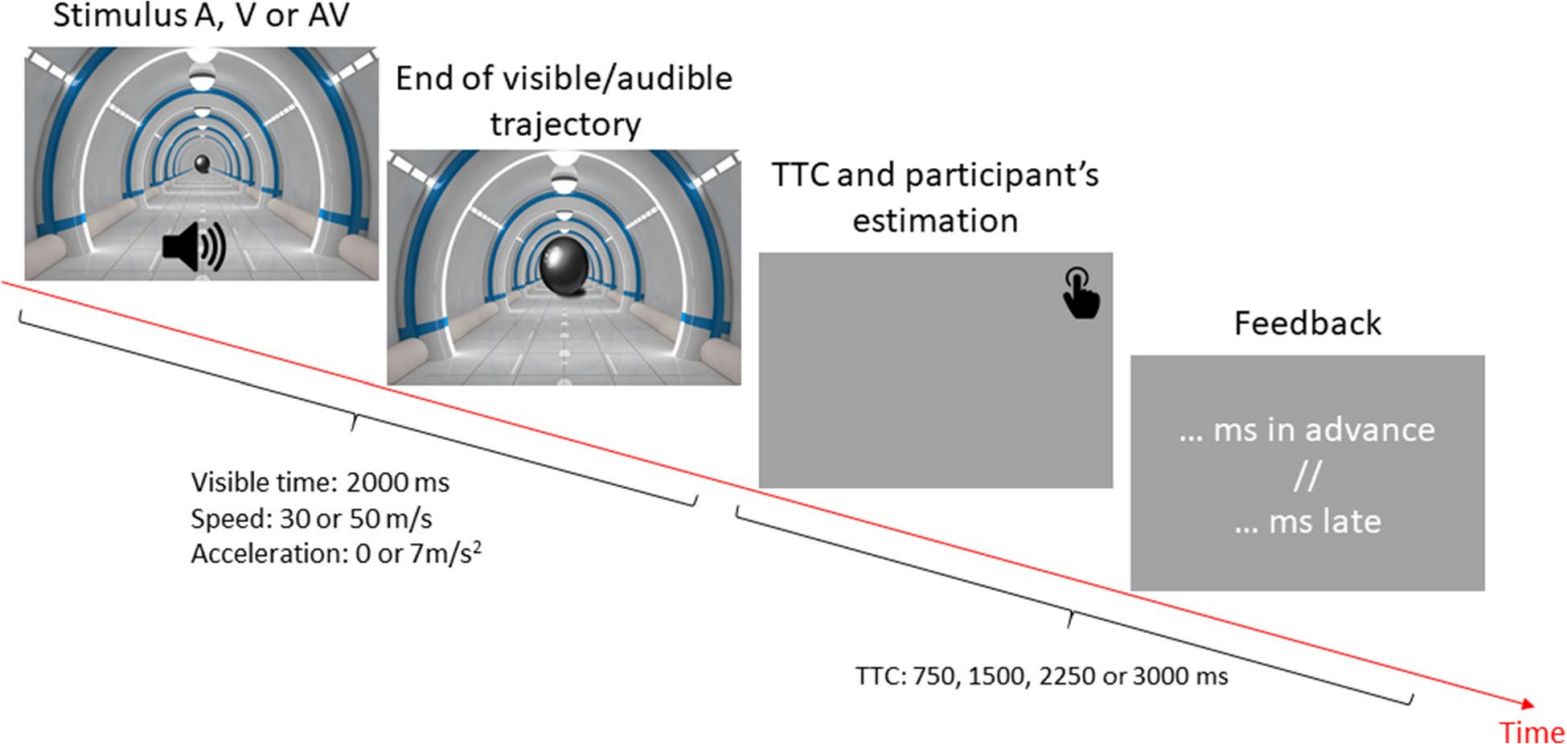
Experimental paradigm. Participants performed a TTC estimation task with visual-only (V), auditory-only (A) or audiovisual (AV) stimuli. On each trial, a ball moving towards the participant was visible for 2000 milliseconds and occluded during its trajectory for a TTC of 750, 1500, 2250 or 3000 milliseconds. The ball could move at a constant or accelerated speed. In the visual-only modality, the perception of motion was induced by changing the size of the ball. In the auditory modality, motion perception was induced by changing the intensity of the complex tone (see Supplementary Materials Table 1 and Table 2 for details)

In this experiment, we tested the TTC estimation performance of participants using visual-only (V), auditory-only (A) and audiovisual (AV) modalities. The stimulus for the TTC estimation task was presented to the participants on a Dell Precision T1700 equipped with a 1.6 GHz i7 processor and a 22-in. screen (resolution 1920 × 1080, dimension 47.6 × 26.8 cm in horizontal by vertical). The visual scene was generated using MATLAB R2019b software. The visual stimulus consisted of a looming ball moving in a corridor (Fig. 1). The ball was on a direct collision course with the participant’s position. Additionally, the visual simulation was perspective-correct, with the object's size and position dynamically adjusted to simulate an accurate approach trajectory in depth. After 2000 ms of visible and/or audible movement, the ball and/or the sound disappeared for a duration of 750, 1500, 2250 or 3000 ms. Participants had to press the space bar on the keyboard to indicate the estimated arrival time. After each trial, participants received feedback on their performance: “early” or “too late” and the error in ms (difference between the estimated and actual arrival times). The ball could move at a speed of 30 or 50 m/s and at a constant speed (acceleration of 0 m/s^2^) or at an acceleration of 7 m/s^2^. The initial speeds were 30 m/s and 50 m/s. For the constant speed trials, this speed remained the same throughout the visible period. For the trials with an acceleration of 7 m/s^2^, given that the visible time duration was 2 seconds, the speed at the moment of occlusion increased by 14 m/s. Thus, the speeds at occlusion were 44 m/s (from an initial speed of 30 m/s) or 64 m/s (from an initial speed of 50 m/s) (Supplementary material: Table 1 and 2).

Variations in speed and acceleration prevented a perfect correlation between TTC and occlusion distance. The use of variable speeds, accelerations and TTC led to a variation in the starting position and angular size of the ball, but did not allow participants to deduce the TTC from it (Baurès et al., 2021).

In the A and AV condition, the audio stimulus was a stationary sound. It consisted of a complex tone made up of three frequencies: a fundamental frequency of 498 Hz, which determined the perceived pitch of the sound and two harmonics at 1003 Hz and 1502 Hz, which enriched the timbre of the sound.

The frequency remained constant during the movement, with only the sound intensity varying in relation to the simulated distance, thus creating the illusion of motion (Supplementary material: Table 1 and 2). The sounds were presented through wired loudspeakers (Companion® 2 series III) positioned on either side of the screen, approximately 1 meter away from the participant. The auditory stimuli correspond to the approach of the sound source in an auditory free field, meaning there were no reflective surfaces present in the simulated scenario.

The sound intensity at 0.5 m (never reached because the ball disappeared before reaching the observer) was set at 120 dB. A Steinberg System SBS-SM-130C sound level meter was employed for measuring and calibrating sound levels. Given two distances d_1_ and d_2_ with d_1_>d_2_, the sound intensity varied as a function of:

$${\mathrm{dB}}_{2}={\mathrm{dB}}_{1}+20 {\mathrm{log}}_{10}\left({\mathrm{d}}_{1}/{\mathrm{d}}_{2}\right)$$

with

dB_1_: Sound intensity at distance
d_1_ and dB_2_: Sound intensity at distance d_2_

The sounds were generated using Max 8 software.

In the auditory-only condition, the corridor was empty, the ball was only audible but not visible.

The 48 different conditions (4 TTC × 2 Speeds x 2 Accelerations x 3 Modalities) were repeated once per block, and participants performed twelve blocks (12 repetitions of each trial). The trials were randomised, presented to the participants, and the participants' response times were recorded using eventIDE software.

### Data analysis

Statistical analysis of the data was carried out using R software.

For each trial, we computed the constant error (CE), corresponding to the mean difference between the estimated and actual arrival times. A positive CE indicates an overestimation of the TTC (i.e., participants respond too late), on the contrary, a negative CE indicates an underestimation of the TTC (i.e., participants respond too early). To exclude extreme data points, we applied a Tukey criterion to the data collected per combination of participant and experimental condition, excluding 2.66% of the trials (299 of a total of 11 221 trials), which were 1.5 interquartile ranges below the first or above the third quartile.

The CE was then analysed using a type 3 repeated-measures ANOVA provided by the ezANOVA function in R (ez package), with the factors Acceleration, Modality, and TTC being within-subject factors. Post-hoc t-tests with Hochberg correction were carried out to localize the differences when the main test was significant. Additionally, we calculated Cohen’s d_z_ (Cohen, 1988) to quantify the effect size.

We also carried out a repeated measures correlation following Bland & Altman’s method (1995), examining the relationship between the Mean CE and TTC for each Modality and each level of Acceleration. This method allows us to assess the association between these variables while accounting for the within-subject design.

## Results

We assessed whether there was an effect of modality on participants' TTC estimation performance and whether this effect was the same for each TTC and independent of the Acceleration condition.

**Fig. 2:**
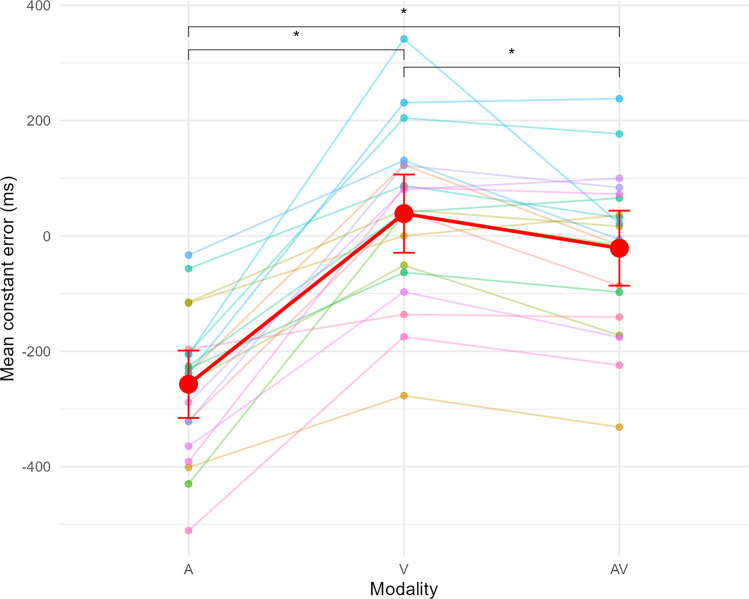
Mean CE as a function of Modality (A=Auditory-only, V=Visual-only, AV=Audiovisual). Points and lines with the same colour belong to the same participant. The red dots correspond to the average of CE. Error bars indicate the 95% confidence interval (CI95). * indicates a significant difference at the *p* < 0.05 threshold

The ANOVA analysing the CE showed a significant effect of Modality on CE F(2, 38) = 56.99, p <.001, ${\eta }_{p}^{2}$ = 0.75. The CE differed significantly between the three modalities (Fig. 2). The CE in the A-only modality was underestimated (mean CE_A_ = $-$ 257ms) and went to overestimated in the V-only condition (mean CE_V_ = 65 ms), with the AV modality in-between those values (mean CE_AV_ = $-$ 21.7 ms), slightly closer to the V-only condition however. Effect sizes given by d_z_ were high when comparing the V and A modalities (d_z_ = 2.02) and AV and A modalities (d_z_ = 1.62), and moderate for V and AV modalities (d_z_ = 0.74).

**Fig. 3:**
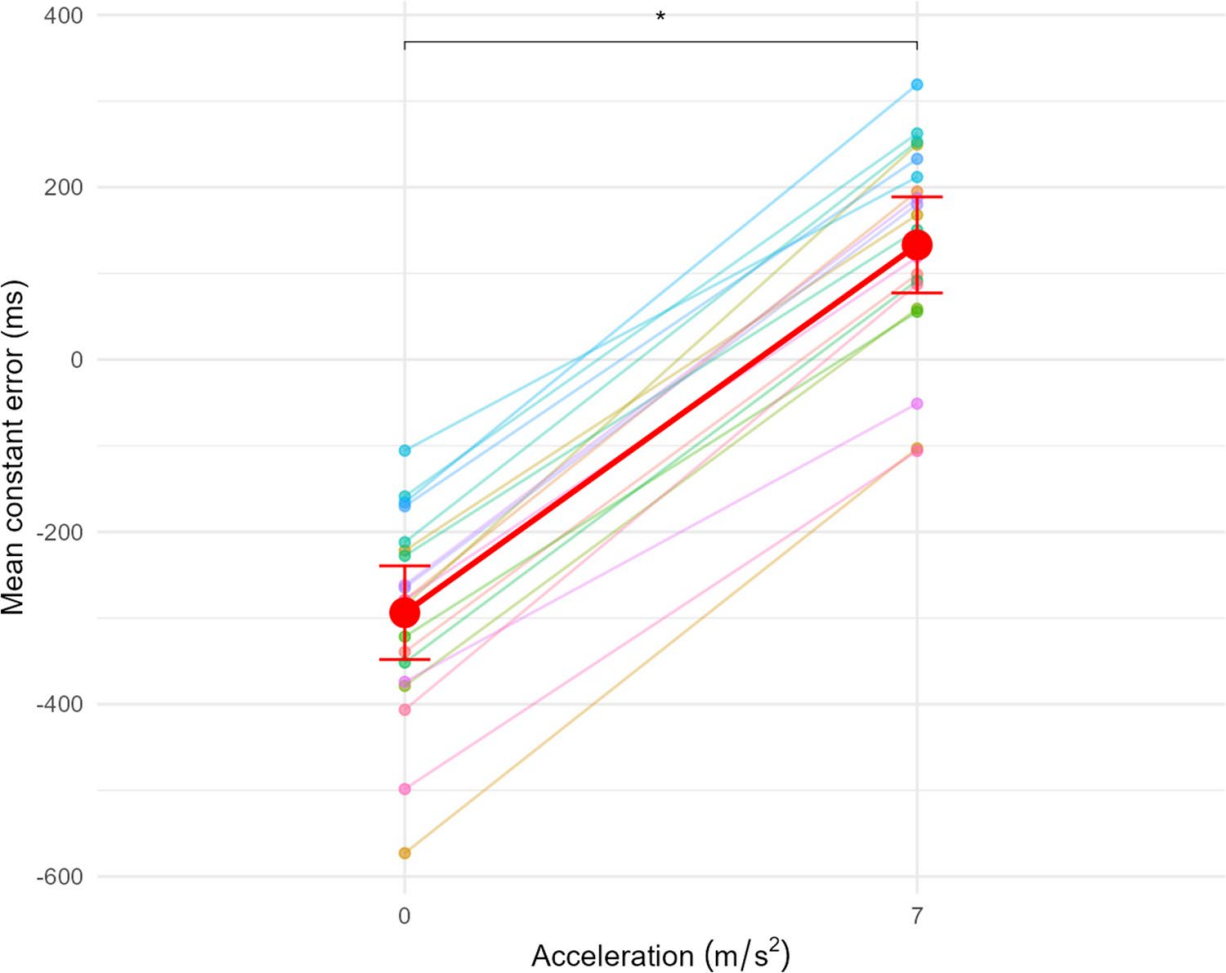
Mean CE as a function of Acceleration. Points and lines with the same colour belong to the same participant. An acceleration of 0 m/s^2^ corresponds to a constant speed. The red dots correspond to the average of CE. Error bars indicate the 95% confidence interval (CI95). * indicates a significant difference at the *p* < 0.05 threshold

The effect of Acceleration on CE was also significant F(1, 19) = 1151.15, p <.001, ${\eta }_{p}^{2}$ = 0.98 (Fig. 3). At constant speed, the CE was significantly underestimated (mean CE_0_ = $-$ 294 ms), whereas at accelerated the CE was significantly overestimated (mean CE₇ = 133 ms). The effect size indicates a very strong difference, with d_z_ = 7.66, suggesting that all participants are strongly affected by the acceleration level.

**Fig. 4:**
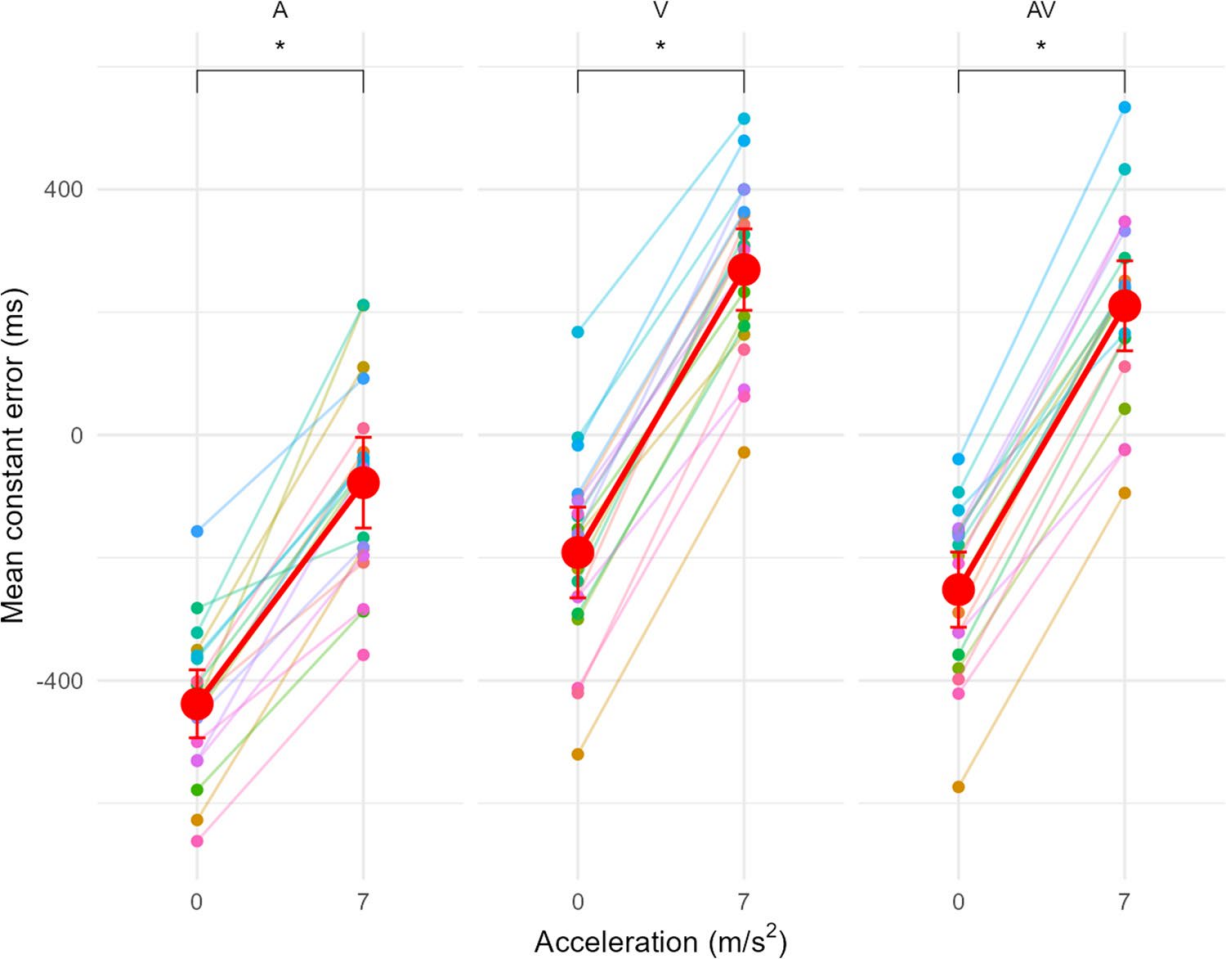
Mean CE as a function of Acceleration for each Modality (A=Auditory-only, V=Visual-only, AV=Audiovisual). Points and lines with the same colour belong to the same participant. An acceleration of 0 m/s^2^ corresponds to a constant speed. The red dots correspond to the average of CE. Error bars indicate the 95% confidence interval (CI95). * indicates a significant difference at the *p* < 0.05 threshold

An interaction was found between the factors Acceleration and Modality, F(2, 38) = 7.73, p <.001, ${\eta }_{p}^{2}$ = 0.30, indicating that the effect of acceleration on constant error (CE) varied depending on the modality (Fig. 4). For all three modalities, under constant speed (0 m/s^2^), there was a consistent underestimation of the TTC. The most pronounced underestimation occurred in the A-only modality (mean CE_A,0_ = $-$ 437 ms), followed by the AV condition (mean CE_AV,0_ = $-$ 252 ms), with the smallest underestimation observed in the V condition (mean CE_V,0_ = $-$ 191 ms). For the V and AV modalities, transitioning to accelerated speed resulted in an overestimation of TTC compared to constant speed, for both V (mean CE_V,7_ = 270 ms) and AV (mean CE_AV,7_ = 210 ms). Similarly, for the A modality, acceleration caused participants to respond later, resulting in a smaller underestimation (mean CE_A,7_ = $-$ 77.4 ms). While all three modalities followed a similar pattern, the overestimation due to acceleration caused a larger difference in CE between constant and accelerated speed for the V (d_z_ = 5.74) and AV (d_z_ = 5.97) modalities compared to the A modality (d_z_ = 2.93). This suggests that acceleration had a smaller impact on the A modality compared to the V and AV conditions, where the shift of the CE toward an overestimation at accelerated speed was more pronounced.

**Fig. 5:**
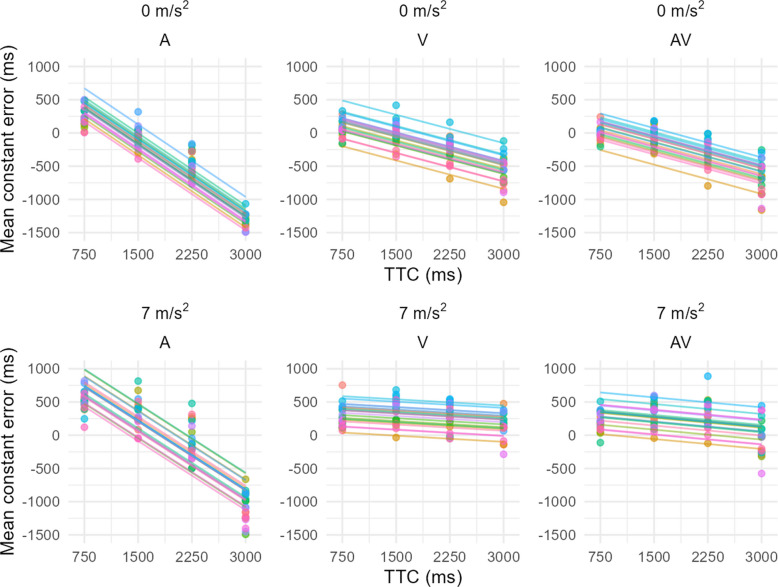
Repeated measures correlation between TTC and CE for the different Acceleration and Modality levels. Points and lines with the same colour belong to the same participant. Panels of the top line correspond to the constant speed condition (Acceleration = 0 m/s^2^) while the lower line panels correspond to the accelerated speed condition (Acceleration = 7 m/s^2^). Panels on the left, middle and right are respectively representing the Audio, Visual and Audiovisual modalities

The ANOVA also revealed a significant effect of TTC F(3, 57) = 324.83, p <.001, ${\eta }_{p}^{2}$ = 0.94. As the TTC increases, the CE becomes more and more underestimated, showing that it is more difficult to estimate the TTC for longer TTCs (Fig. 5).

**Fig. 6:**
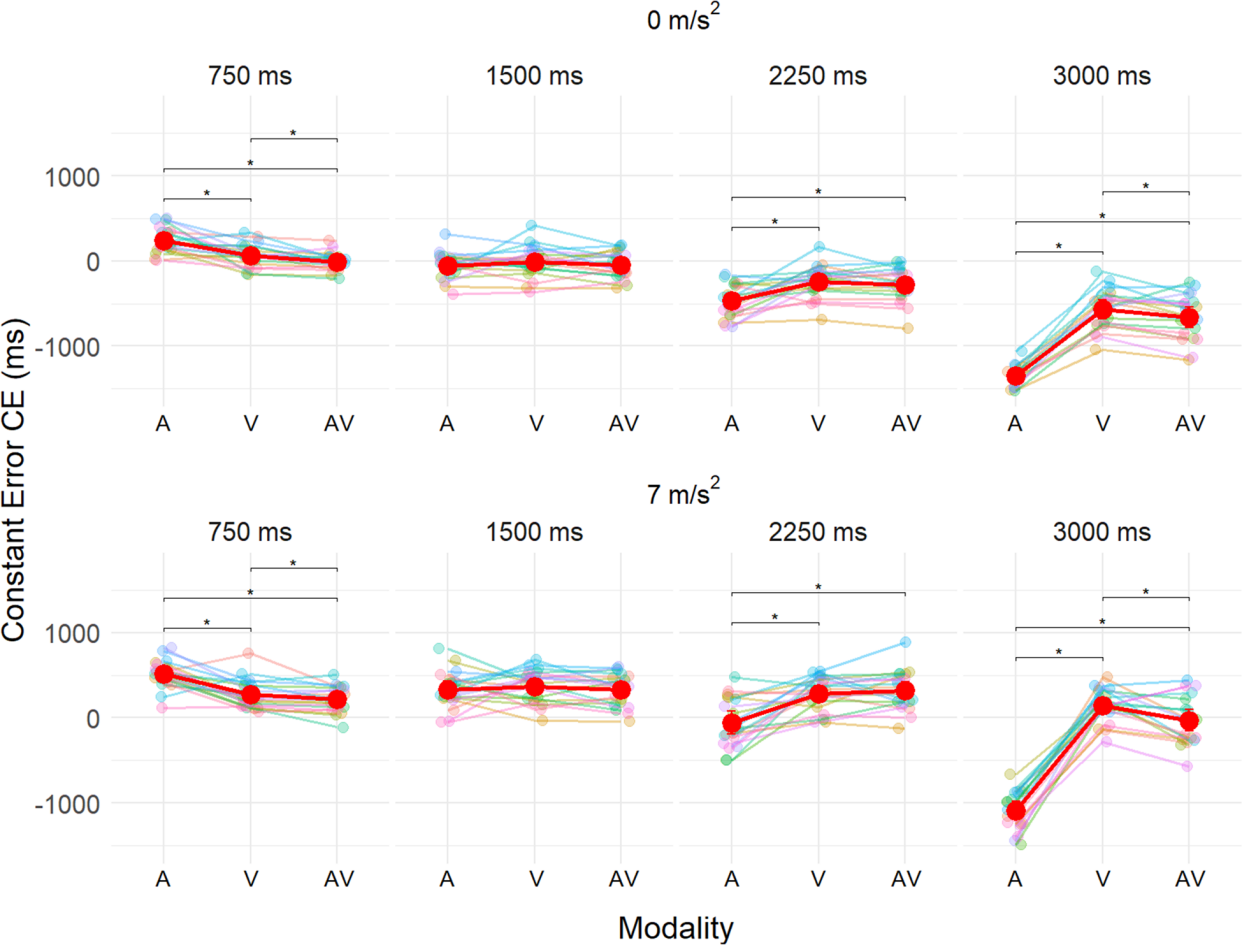
Mean CE as a function of Modality (A=Auditory-only, V=Visual-only, AV=Audiovisual). Points and lines with the same colour belong to the same participant. The red dots correspond to the average of CE. Panels of the top line correspond to the constant speed condition (Acceleration = 0 m/s^2^) while the lower line panels correspond to the accelerated speed condition (Acceleration = 7 m/s^2^). TTC varies from left to right panels to represent the 750, 1500, 2250, 3000 ms levels. * indicates a significant difference at the *p* < 0.05 threshold

There was also a significant effect of the interaction between these three factors F(6, 114) = 8.98, p <.001, ${\eta }_{p}^{2}$ = 0.32. Post hoc tests showed that at both the shortest (750 ms) and longest (3000 ms) TTC, the constant error (CE) in the A condition was more pronounced compared to the V and AV conditions, either overestimated (short TTC) or underestimated (long TTC). Additionally, at these TTC, significant differences in CE were observed between the V and AV conditions, although the magnitude of the difference was smaller than that observed for the A condition. For the intermediate TTC (1500 and 2250 ms), little to no difference was observed between the conditions, likely because the CE transitioned from positive to negative at these levels (Fig. 6). Importantly, these same patterns were observed under both constant and accelerated speed conditions with a shift in values towards positive values under accelerated speed, due to the overestimation caused by acceleration. See supplementary material : Table 3 and 4, for details of CE and significant results.

The results described above suggest that the relationship linking TTC to the constant error had very different parameters in the A condition compared to V and AV. To evaluate this hypothesis, we used repeated measures correlation as advised by Bland & Altman (1995) in each of the conditions (Fig. 5).

Under constant speed conditions, in the A condition, we observed a significant correlation between the CE and the TTC (r^2^ = 0.86): as the TTC increased, the CE decreased sharply, transitioning from overestimation to underestimation of the TTC with a slope of $-$ 0.73. In contrast, the correlation between CE and TTC was weaker for the V (r^2^ = 0.41) and AV (r^2^ = 0.53) modalities and the transition from overestimation to underestimation was less pronounced with slopes of $-$ 0.28 and $-$ 0.29 for V and AV respectively.

Under accelerated speed conditions, the pattern for the A condition remained nearly identical to that observed under constant speed. We still observed a significant correlation between the CE and the TTC (r^2^ = 0.77), with a slope of $-$ 0.69. In the V and AV conditions, however, the correlation between CE and TTC became again much weaker (r^2^ = 0.41 and 0.28 respectively). The slope of the regression line approached zero ($-$ 0.06 for V, $-$ 0.10 for AV), indicating that CE became almost independent of TTC under acceleration.

## Discussion

This study partially confirms our hypothesis. Our results show that observers do use both modalities in the AV condition and therefore demonstrate a multisensory integration, but for both levels of acceleration and better performance is not always observed in the multimodal condition.

In the A-only condition, the TTC is underestimated for both acceleration levels. In the V-only condition, the TTC estimation is either underestimated or overestimated depending on the acceleration levels, but in both cases above the A-only modality. In-line with this unimodal error patterns, the error in the AV condition appears to be in-between these two performances levels, however closer to the V-only level than the A-only level. The results are congruent with the idea that the underestimation caused by the A cues lead the participant to respond earlier in the AV condition and therefore to underestimate the TTC compared to the V-only condition. Therefore, when the visual estimation is overestimated (i.e., CE > 0) the A cues improve performance by bringing the CE closer to zero. On the other hand, when the V estimation is underestimated (i.e., CE < 0), the auditory cues increase this underestimation even more. Whether multisensory integration leads to an overestimation or underestimation of TTC compared to single modality conditions therefore, depends on the level of baseline CE in A-only and V-only conditions. This result is in line with those of Schiff and Oldak (1990) who show multisensory integration in TTC estimation, but this does not always seem to be beneficial as the best results are obtained in V only in their study at constant speed. Here we do observe multisensory integration only for the most extreme TTC. Indeed, for short TTCs, the TTC being overestimated, audiovisual multisensory integration has a beneficial effect while for the longest TTCs, the TTC being underestimated, audiovisual multisensory integration has a detrimental effect. For intermediate TTC, there does not appear to be any multisensory integration, with V and AV performance being identical. This result may be explained by already optimal visual performance in V, leading to ignorance of auditory cues.

For the A modality, only the sound intensity was varied to signal the TTC level. A strong correlation with a highly negative slope was observed between CE and TTC in the A condition. This suggests that sound intensity strongly influences the TTC estimation, both at constant and accelerated speed. In the V and AV modalities however, the relationship is weaker at constant speed and almost cancelled at accelerated speed. This suggests that in the presence of visual cues, auditory cues are almost entirely disregarded. It would have been interesting to test incongruent situations, as done in the studies by DeLucia et al. (2016) and Keshavarz et al. (2017). This approach would have allowed us to apply the Fetsch et al. (2012) model to calculate the perceptual weights of each modality, providing valuable insights into whether the weight of auditory cues differs between constant and accelerated speeds.

We hypothesised that performance in AV would be identical to that in V at constant speed (DeLucia et al., 2016; Keshavarz et al., 2017) and that multisensory integration would only be observed at accelerated speed (Wessels et al., 2020a, 2020b). These hypotheses are not confirmed in our study. In fact, the significant variations in performance observed for the extreme TTCs (the longest and shortest) are present at both constant and accelerated speeds. Nevertheless, if multisensory integration is present for the two levels of acceleration, its impact on TTC estimation varies depending on the acceleration. At accelerated speed, TTC was overestimated. So, particularly in this context of accelerated speed, auditory cues, leading to an underestimation, are beneficial, partially correcting the visual overestimation. Thus, the auditory cues tend to correct the visual overestimation caused by acceleration. However, the audiovisual estimation at accelerated speed remains overestimated compared to the estimation at constant speed, showing that the multimodal nature of the stimulus does not fully compensate for the visual overestimation due to acceleration. We showed that acceleration strongly influences CE across all three modalities, leading to a shift from underestimation to overestimation in the V and AV conditions. Although the difference between constant and accelerated speed is significant for all three modalities, the Cohen’s dz for the A modality is lower, indicating that acceleration has a smaller impact on CE in A. This result supports the idea that acceleration is better perceived in the A modality compared to V and AV. While multisensory integration is known to improve perception and performances in general (Cappe et al., 2009; Giard & Peronnet, 1999), the variability of multisensory effects has already been shown in other tasks and depend on several factors (Juan et al., 2017). Thus, there are a variety of sources of variability in multisensory integration that can lead to changes in behavioural performance. The cognitive level required by the task, as well as the physical and semantic parameters of the stimuli, can have a major influence on performance.

Several possible explanations could account for our results. One possibility is that our hypothesis regarding acceleration was correct, but our protocol failed to effectively demonstrate it. There are two primary reasons for this. First, participants may have been unable to detect the acceleration, potentially due to sound parameters that were not sufficiently aligned with real-world dynamics. As noted in previous studies, for example Oberfeld et al. (2022) or Wessels et al. (2022a, 2022b) a virtual reality paradigm could be beneficial to better match the natural context and provide more realistic sensory cues. It is important to note that the previous studies on acceleration, such as those mentioned in our introduction (Keshavarz et al. 2017; DeLucia et al. 2016), presented sounds that signalled the presence of acceleration, particularly in relation to vehicles with combustion engines. In these studies, acceleration was signalled by the change in engine sound, which is directly related to the vehicle's engine rotational speed increasing during acceleration. In contrast, our study used a simplified sound design where only the intensity of the sound varied, simulating an approaching sound source in an auditory free field (i.e., no reflective surfaces were present). This setup likely created a mismatch between the visually presented tunnel and the auditory stimuli, potentially reducing the effectiveness of our approach in signalling acceleration. The absence of realistic auditory cues that would typically signal acceleration in naturalistic scenarios may have contributed to the difficulty in detecting and responding to acceleration, leading to the absence of the expected multisensory integration effects. Our study sought to explore a more simplified and generalizable scenario where auditory cues were limited to variations in sound intensity with distance, which would apply to a wider range of objects, not just those with engine-generated sounds. This choice however may have contributed to the low impact of the multisensory condition.

Another explanation could come from the distribution of trials within our experimental blocks. Trials with and without acceleration were presented randomly in the same block. It is possible that, following the feedback, participants averaged the level of acceleration by considering an intermediate level of acceleration for all trials, even those at constant speed. This would result in identical performance at constant and accelerated speeds. The study of Otto & Mamassian (2012) used a stimulus detection task in the A, V and AV modalities and analysed participants' reaction times (RT). The authors showed that the latency of response to one trial had an influence on the latency of the next trial, indicating an effect of trial history. Transposed to our experiment, our participants may have averaged the acceleration levels and therefore decrease the specific advantage of the multisensory condition in the accelerated condition. It would therefore be interesting to test only one level of acceleration per block in order to limit the influence of the previous trial, especially if two trials with different levels of acceleration were presented in succession.

In conclusion, we demonstrate here a multisensory integration when estimating the TTC of an audiovisual object. This integration holds whether the object moves at constant or accelerated speed, on the contrary to our initial hypothesis. The AV performance is always intermediate between the A and the V performances, yet closer to the V. As such, the multisensory impact depends on the baseline level of the unimodal conditions.

## Supplementary Information

Below is the link to the electronic supplementary material.

ESM1 (DOCX 29.6 KB)

ESM2 (DOCX 29.5 KB)

ESM3 (DOCX 32.0 KB)

ESM4 (DOCX 13.3 KB)

## Data Availability

Data that support the findings of this study are available on this link: https://osf.io/krjy8/?view_only=ff58d7f9f17549a7b6c48f58075dfa1d

## Code availability

R code for statistical analysis that support the findings of this study is available on this link: https://osf.io/krjy8/?view_only=ff58d7f9f17549a7b6c48f58075dfa1d
