# Supplementary material for: Audiovisual estimation of Time-to-contact
Source: Atten Percept Psychophys. 2026 Jan 13;88(2):51. doi: 10.3758/s13414-025-03176-6 (PMC12795859; doi:10.3758/s13414-025-03176-6)
Supplement: Supplementary file 1 — (DOCX 29.6 KB) [file 13414_2025_3176_MOESM1_ESM.docx]

|  | | Acceleration 0 m/s^2^ | | | | | | | |
| --- | --- | --- | --- | --- | --- | --- | --- | --- | --- |
|  |  | Speed 30 m/s | | | | Speed 50 m/s | | | |
|  |  | TTC (s) | | | | TTC (s) | | | |
|  |  | 0.75 | 1.5 | 2.25 | 3 | 0.75 | 1.5 | 2.25 | 3 |
| Angular size (deg) | First frame | 2.50 | 1.96 | 1.62 | 1.37 | 1.50 | 1.18 | 0.97 | 0.82 |
|  | Last visible frame | 9.14 | 4.58 | 3.05 | 2.29 | 5.49 | 2.75 | 1.83 | 1.37 |
| Sound intensity (dB) | First frame | 61.67 | 59.57 | 57.89 | 56.48 | 57.24 | 55.14 | 53.45 | 52.04 |
|  | Last  visible frame | 72.84 | 66.93 | 63.41 | 60.91 | 68.51 | 62.49 | 58.97 | 56.47 |
| Distance (m) | First frame | 82.50 | 105.00 | 127.50 | 150.00 | 137.50 | 175.00 | 212.50 | 250.00 |
|  | Last  visible frame | 22.53 | 45.03 | 67.53 | 90.03 | 37.55 | 75.05 | 112.55 | 150.05 |
| Speed  (m/s) | First frame | 30 | 30 | 30 | 30 | 50 | 50 | 50 | 50 |
|  | Last  visible frame | 30 | 30 | 30 | 30 | 50 | 50 | 50 | 50 |

Table 1
