# Supplementary material for: Audiovisual estimation of Time-to-contact
Source: Atten Percept Psychophys. 2026 Jan 13;88(2):51. doi: 10.3758/s13414-025-03176-6 (PMC12795859; doi:10.3758/s13414-025-03176-6)
Supplement: Supplementary file 2 — (DOCX 29.5 KB) [file 13414_2025_3176_MOESM2_ESM.docx]

|  | | Acceleration 7 m/s^2^ | | | | | | | |
| --- | --- | --- | --- | --- | --- | --- | --- | --- | --- |
|  |  | Speed 30 m/s | | | | Speed 50 m/s | | | |
|  |  | TTC (s) | | | | TTC (s) | | | |
|  |  | 0.75 | 1.5 | 2.25 | 3 | 0.75 | 1.5 | 2.25 | 3 |
| Angular size (deg) | First frame | 1.89 | 1.39 | 1.08 | 0.87 | 1.26 | 0.95 | 0.75 | 0.61 |
|  | Last  visible frame | 5.89 | 2.79 | 1.77 | 1.26 | 4.12 | 1.98 | 1.27 | 0.92 |
| Sound intensity (dB) | First frame | 59.25 | 56.60 | 54.39 | 52.49 | 55.70 | 53.23 | 51.19 | 49.43 |
|  | Last  visible frame | 69.11 | 62.62 | 58.65 | 55.72 | 66.01 | 59.66 | 55.82 | 53.01 |
| Distance (m) | First frame | 108.97 | 147.87 | 190.72 | 237.50 | 163.97 | 217.87 | 275.72 | 337.50 |
|  | Last  visible frame | 35.01 | 73.92 | 116.76 | 163.54 | 50.03 | 103.94 | 161.78 | 223.56 |
| Speed  (m/s) | First frame | 30 | 30 | 30 | 30 | 50 | 50 | 50 | 50 |
|  | Last  visible frame | 44 | 44 | 44 | 44 | 64 | 64 | 64 | 64 |

Table 2
