# Supplementary material for: Audiovisual estimation of Time-to-contact
Source: Atten Percept Psychophys. 2026 Jan 13;88(2):51. doi: 10.3758/s13414-025-03176-6 (PMC12795859; doi:10.3758/s13414-025-03176-6)
Supplement: Supplementary file 3 — (DOCX 32.0 KB) [file 13414_2025_3176_MOESM3_ESM.docx]

|  | Acceleration 0 m/s^2^ | | | | | | |
| --- | --- | --- | --- | --- | --- | --- | --- |
|  | TTC 750 ms | | |  | TTC 1500 ms | | |
|  | CE_A750_ = 243.77 | CE_V750_ = 66.09 | CE_AV750_ =  -8.87 |  | CE_A1500_ =  -56.62 | CE_V1500_ =  -10.21 | CE_AV1500_ =  -45.78 |
| CE_A750_ = 243.77 |  | * | * | CE_A1500_ =  -56.62 |  | ns | ns |
| CE_V750_ = 66.09 | * |  | * | CE_V1500_ =  -10.21 | ns |  | ns |
| CE_AV750_ =  -8.87 | * | * |  | CE_AV1500_ =  -45.78 | ns | ns |  |
|  |  | | | | | | |
|  | TTC 2250 ms | | |  | TTC 3000 ms | | |
|  | CE_A2250_ =  -469.77 | CE_V2250_ =  -246.43 | CE_AV2250_ =  -281.67 |  | CE_A3000_ =  -1437.27 | CE_V3000_ =  -567.05 | CE_AV3000_ =  -666.79 |
| CE_A2250_ =  -469.77 |  | * | * | CE_A3000_ =  -1437.27 |  | * | * |
| CE_V2250_ =  -246.43 | * |  | ns | CE_V3000_ =  -567.05 | * |  | * |
| CE_AV2250_ =  -281.67 | * | ns |  | CE_AV3000_ =  -666.79 | * | * |  |
|  |  | | | | | | |
|  | Acceleration 7 m/s^2^ | | | | | | |
|  | TTC 750 ms | | |  | TTC 1500 ms | | |
|  | CE_A750_ =  513.50 | CE_V750_ = 272.86 | CE_AV750_ =  219.95 |  | CE_A1500_ =  335.27 | CE_V1500_ =  371.59 | CE_AV1500_ =  326.03 |
| CE_A750_ =  513.50 |  | * | * | CE_A1500_ =  335.27 |  | ns | ns |
| CE_V750_ = 272.86 | * |  | * | CE_V1500_ =  371.59 | ns |  | ns |
| CE_AV750_ =  219.95 | * | * |  | CE_AV1500_ =  326.03 | ns | ns |  |
|  |  | | | | | | |
|  | TTC 2250 ms | | |  | TTC 3000 ms | | |
|  | CE_A2250_ =  -58.15 | CE_V2250_ =  288.08 | CE_AV2250_ =  323.97 |  | CE_A3000_ =  -1087.67 | CE_V3000_ =  149.386 | CE_AV3000_ =  -27.14 |
| CE_A2250_ =  -58.15 |  | * | * | CE_A3000_ =  -1087.67 |  | * | * |
| CE_V2250_ =  288.08 | * |  | ns | CE_V3000_ =  149.386 | * |  | * |
| CE_AV2250_ =  323.97 | * | ns |  | CE_AV3000_ =  -27.14 | * | * |  |

Table 3
