# Supplementary material for: Audiovisual estimation of Time-to-contact
Source: Atten Percept Psychophys. 2026 Jan 13;88(2):51. doi: 10.3758/s13414-025-03176-6 (PMC12795859; doi:10.3758/s13414-025-03176-6)
Supplement: Supplementary file 4 — (DOCX 13.3 KB) [file 13414_2025_3176_MOESM4_ESM.docx]

| Predictor | *df_Num_* | *df_Den_* | *Epsilon* | *F* | *p* | $\eta_{p}^{2}$ |
| --- | --- | --- | --- | --- | --- | --- |
| Acceleration | 1.00 | 19.00 |  | 1151.15 | .000 | .98 |
| Modality | 1.52 | 28.91 | 0.76 | 56.99 | .000 | .75 |
| TTC | 1.67 | 31.81 | 0.56 | 324.83 | .000 | .94 |
| Acceleration x Modality | 1.55 | 29.44 | 0.77 | 8.01 | .000 | .30 |
| Acceleration x TTC | 3.27 | 62.06 | 1.09 | 52.02 | .000 | .73 |
| Modality x TTC | 3.04 | 57.69 | 0.51 | 142.64 | .000 | .88 |
| Acceleration x Modality x TTC | 5.67 | 107.66 | 0.94 | 8.98 | .000 | .32 |

Table 4
